# Cost-effectiveness analysis of first-line cadonilimab plus chemotherapy in HER2-negative advanced gastric or gastroesophageal junction adenocarcinoma

**DOI:** 10.3389/fimmu.2025.1575627

**Published:** 2025-05-13

**Authors:** Zhifeng Zhou, Yanqing Yang, Shaofang Chen, Maojin You

**Affiliations:** ^1^ Department of Pharmacy, Fuzhou University Affiliated Provincial Hospital, Fuzhou, Fujian, China; ^2^ Department of Clinical Nutrition, Zhangzhou Affiliated Hospital of Fujian Medical University, Zhangzhou, Fujian, China; ^3^ Department of Pharmacy, Mindong Hospital Affiliated to Fujian Medical University, Ningde, Fujian, China

**Keywords:** cadonilimab, chemotherapy, cost-effectiveness, first-line treatment, HER2-negative, gastric or gastroesophageal junction adenocarcinoma

## Abstract

**Background:**

The COMPASSION-15 trial demonstrated that cadonilimab plus chemotherapy (CAD-CHM) confers clinical benefits over placebo plus chemotherapy (PLA-CHM) as a first-line treatment for human epidermal growth factor receptor 2 (HER2)-negative advanced gastric or gastroesophageal junction (G/GEJ) adenocarcinoma. However, the introduction of cadonilimab substantially elevates treatment costs, and its cost-effectiveness relative to PLA-CHM remains undetermined. This study evaluates the cost-effectiveness of CAD-CHM compared with PLA-CHM from the perspective of the Chinese healthcare system.

**Methods:**

A Markov model with three health states was developed to assess the cost-effectiveness of CAD-CHM in HER2-negative advanced G/GEJ adenocarcinoma. Clinical efficacy data were sourced from the COMPASSION-15 trial, while drug costs were calculated based on national tender prices, and additional costs and utility values were extracted from published literature. The analysis encompassed the overall population, as well as subgroups stratified by programmed death ligand 1 (PD-L1) combined positive score (CPS) ≥ 5 and CPS < 5. Outcomes included total costs, quality-adjusted life-years (QALYs), and incremental cost-effectiveness ratios (ICERs). Sensitivity analyses were conducted to evaluate model robustness.

**Results:**

The ICER of CAD-CHM was $67,378.09 per QALY in the overall population, $48,433.34 per QALY in the PD-L1 CPS ≥ 5 subgroup, and $78,463.86 per QALY in the PD-L1 CPS < 5 subgroup. Key determinants influencing model outcomes included patient weight, cadonilimab cost, and the utility value of progression-free survival. Across all groups, CAD-CHM resulted in an ICER exceeding the willingness-to-pay threshold of $41,511 per QALY, with a 0% probability of cost-effectiveness compared with PLA-CHM.

**Conclusion:**

From the perspective of the Chinese healthcare system, CAD-CHM is not cost-effective as a first-line treatment for HER2-negative advanced G/GEJ adenocarcinoma, either in the overall population or in subgroups stratified by PD-L1 CPS status, compared with chemotherapy alone.

## 1 Introduction

Gastric cancer, including gastroesophageal junction (GEJ) cancer, remains a major global health challenge, ranking fifth in both incidence and mortality among malignant tumors. In 2022, over 968,000 new cases and nearly 660,000 deaths were reported worldwide (1, 2). China bears a disproportionately high gastric or GEJ (G/GEJ) burden, accounting for 42% of global new cases and 45% of gastric cancer-related deaths (3). Due to the lack of specific clinical symptoms, nearly 90% of patients with G/GEJ cancer are diagnosed at an advanced stage (4), resulting in a dismal prognosis, with a five-year survival rate below 5% (5). Adenocarcinoma is the predominant histological subtype, representing over 90% of G/GEJ cancers, and the majority of these tumors are human epidermal growth factor receptor 2 (HER2)-negative (6, 7). For decades, platinum- and fluorouracil-based combination chemotherapy has remained the standard first-line treatment for HER2-negative advanced G/GEJ adenocarcinoma (8). However, therapeutic outcomes remain suboptimal, with a median survival of less than one year (9).

Recent clinical trials have demonstrated that combining programmed cell death protein 1 (PD-1) inhibitors with chemotherapy improves survival in patients with HER2-negative G/GEJ adenocarcinoma (10–14). Moreover, evidence suggests that dual blockade of PD-1 and cytotoxic T-lymphocyte-associated protein 4 (CTLA-4) enhances antitumor responses across multiple solid tumors (15–17). Cadonilimab, a tetravalent bispecific human antibody targeting PD-1 and CTLA-4, exhibits enhanced binding activity in tumor tissues (18–20). The COMPASSION-15 phase III trial recently evaluated the efficacy and safety of cadonilimab plus chemotherapy (CAD-CHM) as a first-line treatment for HER2-negative advanced G/GEJ adenocarcinoma (21). The results demonstrated a significant improvement in median overall survival (OS) (14.1 vs. 11.1 months) and median progression-free survival (PFS) (7.0 vs. 5.3 months) compared with placebo plus chemotherapy (PLA-CHM), with manageable safety. These findings suggest CAD-CHM as a potential first-line treatment option for HER2-negative advanced G/GEJ adenocarcinoma.

Despite its promising clinical efficacy, the incorporation of cadonilimab into combination therapy substantially increases treatment costs, particularly drug-related expenses, imposing a significant financial burden. In resource-limited settings such as China, the cost-effectiveness of CAD-CHM remains a critical consideration for clinicians and policymakers. To date, no comprehensive economic evaluation has assessed CAD-CHM as a first-line treatment for HER2-negative advanced G/GEJ adenocarcinoma. The absence of such analyses may hinder its adoption in healthcare systems with constrained resources. Therefore, this study aims to evaluate the cost-effectiveness of CAD-CHM compared with PLA-CHM as a first-line treatment for HER2-negative advanced G/GEJ adenocarcinoma from the perspective of the Chinese healthcare system.

## 2 Methodology

This study was conducted following the Consolidated Health Economic Evaluation Reporting Standards 2022 (**Supplementary Table 1**) (22).

### 2.1 Model development

**Figure 1:**
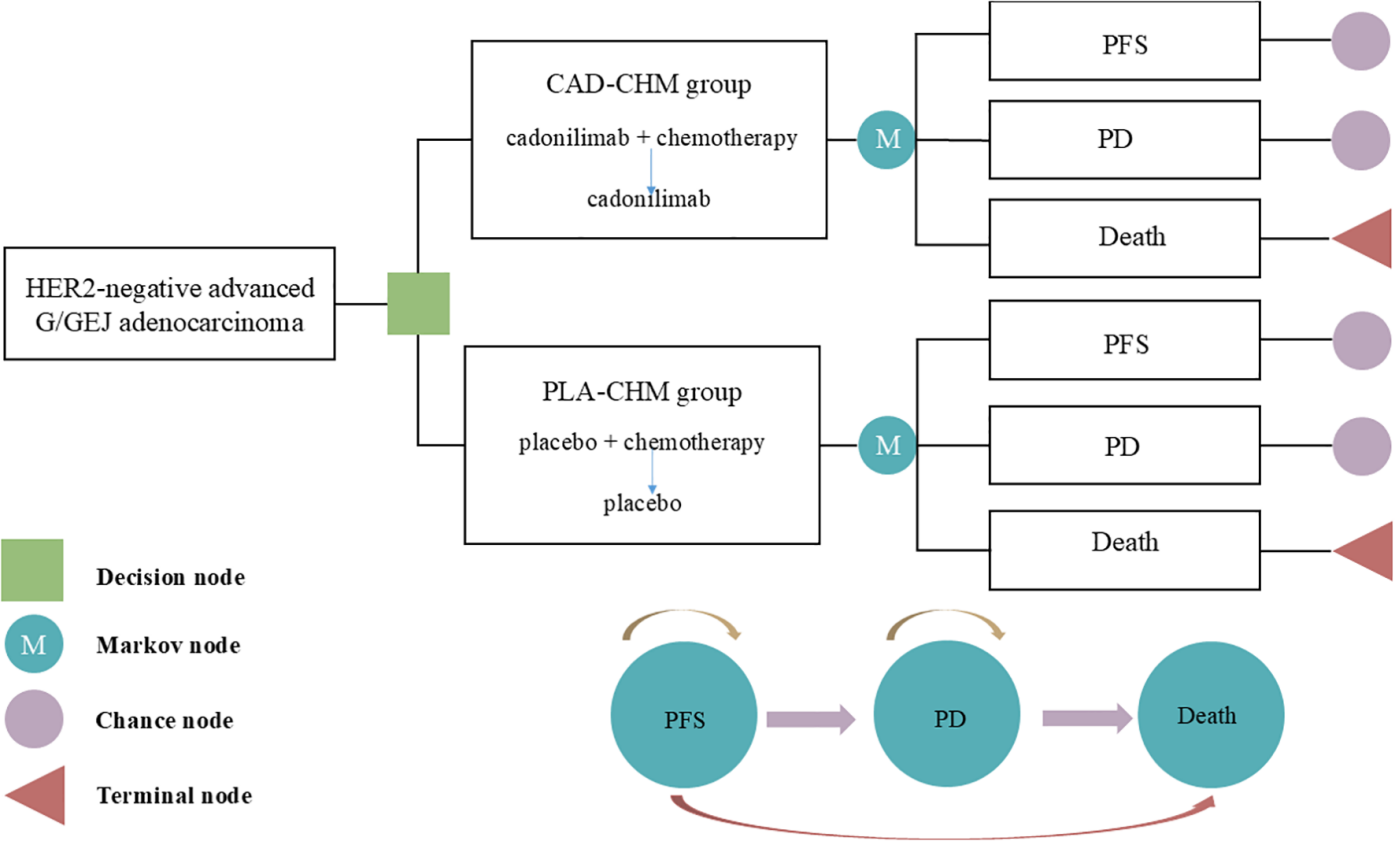
The Markov model simulating outcomes for the COMPASSION-15 trial. All patients started with PFS state and received treatment with CAD-CHM or PLA-CHM. CAD-CHM, cadonilimab plus chemotherapy; G/GEJ, gastric or gastroesophageal junction; HER2, human epidermal growth factor receptor 2; PD, progressive disease; PFS, progression-free survival; PLA-CHM, placebo plus chemotherapy.

A Markov model was developed using TreeAge 2022 software to assess the cost-effectiveness of CAD-CHM versus PLA-CHM as first-line treatments for HER2-negative advanced G/GEJ adenocarcinoma (**Figure 1**). The model comprised three health states: PFS, progressive disease (PD), and death. All patients entered the model in the PFS state, with death as the absorbing state (23). During each cycle, patients could either remain in their current state or transition to the next state, with no possibility of reversal. The cycle length was set at 21 days to align with the treatment cycle, and the model was run for 160 cycles (approximately 9.2 years), by which point 99% of patients were expected to have died. The primary model outcomes included total costs, quality-adjusted life-years (QALYs), and the incremental cost-effectiveness ratios (ICERs). In accordance with the Chinese Guidelines for Pharmacoeconomic Evaluation, the willingness-to-pay (WTP) threshold was set at three times the per capita GDP of China in 2024 ($41,511 per QALY) (24), with an ICER below this threshold considered cost-effective.

### 2.2 Patient clinical treatment data

Clinical treatment data were derived from the COMPASSION-15 trial (21), a multicenter, randomized, phase III trial conducted across 75 hospitals in China. Eligible patients were aged 18–75 years, had histologically confirmed unresectable, locally advanced, or metastatic HER2-negative G/GEJ adenocarcinoma, and had not received prior systemic anticancer therapy. A total of 610 patients were enrolled in the COMPASSION-15 trial, including 256 patients with a PD-L1 Combined Positive Score (CPS) ≥ 5 and 304 patients with a PD-L1 CPS < 5. Patients were randomized to receive either cadonilimab or placebo in combination with chemotherapy (oxaliplatin and capecitabine) (CAD-CHM or PLA-CHM). Specifically, cadonilimab (10 mg/kg) or placebo was administered *via* intravenous infusion on Day 1 of each cycle, oxaliplatin (130 mg/m^2^) *via* intravenous injection on Day 1 of each cycle, and capecitabine (1,000 mg/m^2^) orally twice daily on Days 1–14 of each cycle, with each cycle lasting 3 weeks for up to 6 cycles. Thereafter, patients continued maintenance therapy with cadonilimab or placebo until disease progression or intolerable toxicity. According to the COMPASSION-15 trial (21), the median treatment duration for cadonilimab in the CAD-CHM group was 5.62 months, while oxaliplatin and capecitabine were administered for a median of 4.14 months and 4.17 months, respectively. In the PLA-CHM group, oxaliplatin and capecitabine were administered for a median of 4.14 months. As post-progression treatment details were not provided in the COMPASSION-15 trial, it was assumed that all patients received the best supportive care following disease progression. The study population included the overall population as well as subgroups stratified by PD-L1 CPS (≥ 5 and < 5).

### 2.3 Survival transition probabilities

GetData Graph Digitizer (version 2.26) was used to digitize the PFS and OS curves from the COMPASSION-15 trial. Patient survival data were reconstructed in R software following the method outlined by Guyot et al. (25), and various survival distributions were fitted to extrapolate survival curves beyond the clinical trial follow-up period. The evaluated distributions included exponential, gamma, generalized F, generalized gamma, Gompertz, Weibull, log-logistic, and log-normal models (26, 27). The optimal survival distribution was selected based on Akaike and Bayesian information criteria (28, 29) and subsequently used to estimate transition probabilities between health states (**Supplementary Table 2**). The best-fitting survival distributions and their parameters are detailed in **Table 1**.

**Table 1:** Relevant parameters of survival distribution.

Variable	Value		Source
Survival model for the overall population			
Log-logistic survival model of PFS			
CAD-CHM group	Scale = 0.1263066, Shape = 1.744355		(21)
PLA-CHM group	Scale = 0.2011074, Shape = 2.190154		(21)
Log-logistic survival model of OS			
CAD-CHM group	Scale = 0.06831391, Shape = 1.691376		(21)
PLA-CHM group	Scale = 0.09292127, Shape = 1.952410		(21)
Survival model for the PD-L1 CPS ≥ 5 subgroup			
Log-logistic survival model of PFS			
CAD-CHM group	Scale = 0.1233483, Shape = 1.668463		(21)
PLA-CHM group	Scale = 0.1973213, Shape = 2.321586		(21)
Log-logistic survival model of OS			
CAD-CHM group	Scale = 0.05837011, Shape = 1.482514		(21)
PLA-CHM group	Scale = 0.09334744, Shape = 1.898245		(21)
Survival model for the PD-L1 CPS < 5 subgroup			
Log-logistic survival model of PFS			
CAD-CHM group	Scale = 0.1318699, Shape = 1.857267		(21)
PLA-CHM group	Scale = 0.1999651, Shape = 2.147308		(21)
Log-logistic survival model of OS			
CAD-CHM group	Scale = 0.07192116, Shape = 1.754681		(21)

CAD-CHM, cadonilimab plus chemotherapy; CPS, combined positive score; OS, overall survival; PD-L1, programmed death ligand 1; PFS, progression-free survival; PLA-CHM, placebo plus chemotherapy.

### 2.4 Costs and utilities

This study exclusively considered direct medical costs, including drug expenses, diagnostic tests, routine follow-up, best supportive care, management of grade 3 or higher adverse events with an incidence exceeding 5%, and end-of-life care (**Table 2**). Drug costs were sourced from national tender prices, while other expenditures were obtained from published literature and adjusted to 2024 values using the medical price index from the National Bureau of Statistics of China (30). All costs were reported in US dollars and converted at the 2024 average exchange rate (1 USD = 7.12 CNY). As the COMPASSION-15 trial did not provide quality-of-life data, utility values for PFS and PD were derived from published studies in China (31). To address the potential bias arising from the use of identical utility values for the CAD-CHM and PLA-CHM groups, we considered the disutility of grade 3 or higher adverse events with an incidence exceeding 5% in each treatment group, to improve the accuracy of the health utility values for each treatment group. All costs and utility values were discounted at an annual rate of 5%, in line with pharmacoeconomic guidelines (24). Drug dosages were calculated based on an assumed patient weight of 65 kg and a body surface area of 1.72 m^2^ (32).

**Table 2:** The basic parameters of the input model and the range of sensitivity analyses.

Variable	Base Value	Range		Distribution	Source
		Min	Max		
PLA-CHM group: Incidence of AEs					
Decreased platelet count	0.285	0.228	0.342	Beta	(21)
Decreased neutrophil count	0.151	0.121	0.181	Beta	(21)
Decreased white blood cell count	0.072	0.058	0.086	Beta	(21)
Anemia	0.102	0.082	0.122	Beta	(21)
Hypokalemia	0.059	0.047	0.071	Beta	(21)
PLA-CHM group: Incidence of AEs					
Decreased platelet count	0.250	0.200	0.300	Beta	(21)
Decreased neutrophil count	0.148	0.118	0.178	Beta	(21)
Decreased white blood cell count	0.063	0.050	0.076	Beta	(21)
Anemia	0.125	0.100	0.150	Beta	(21)
Hypokalemia	0.010	0.008	0.012	Beta	(21)
Cost ($)					
Cadonilimab (125mg)	235.96	188.77	283.15	Gamma	(33)
Oxaliplatin (100mg)	32.88	26.30	39.46	Gamma	(33)
Capecitabine (500mg)	0.75	0.60	0.90	Gamma	(33)
Decreased platelet count	1157.50	926.00	1389.00	Gamma	(31)
Decreased neutrophil count	454.71	363.77	545.65	Gamma	(34)
Decreased white blood cell count	211.06	168.85	253.27	Gamma	(35)
Anemia	336.97	269.58	404.36	Gamma	(36)
Hypokalemia	3003.00	2402.40	3603.60	Gamma	(37)
Best supportive care per cycle	182.41	145.93	218.89	Gamma	(38)
Routine follow-up per cycle	73.79	59.03	88.55	Gamma	(38)
Diagnostic tests per cycle	357.70	286.16	429.24	Gamma	(34)
End-of-life care	1491.00	1192.80	1789.19	Gamma	(27)
Utility value					
PFS	0.797	0.638	0.956	Beta	(31)
PD	0.577	0.462	0.692	Beta	(31)
Disutility due to AEs					
Decreased platelet count	0.02	0.016	0.024	Beta	(39)
Decreased neutrophil count	0.20	0.160	0.240	Beta	(40)
Decreased white blood cell count	0.20	0.160	0.240	Beta	(40)
Anemia	0.07	0.056	0.084	Beta	(41)
Hypokalemia	0.03	0.024	0.036	Beta	(37)
Discount rate	0.05	0.08	0.00	Fixed	(24)
Weight (kg)	65	52	78	Normal	(31)

AE, adverse event; CAD-CHM, cadonilimab plus chemotherapy; OS, overall survival; PD, progressive disease; PFS, progression-free survival; PLA-CHM, placebo plus chemotherapy.

### 2.5 Sensitivity analysis

A one-way sensitivity analysis was performed to evaluate the impact of parameter variations on model outcomes. Each parameter was adjusted within its reported 95% confidence interval, and where unavailable, a ±20% range around the baseline value was applied. The discount rate was varied from 0% to 8% (**Table 2**). The results were visualized using a tornado diagram. To assess the combined effect of parameter uncertainty, a probabilistic sensitivity analysis was conducted using 1,000 Monte Carlo simulations, with each parameter assigned a specific probability distribution (**Table 2**). The results were illustrated in a scatter plot. Additionally, the ICER of CAD-CHM compared with PLA-CHM was iteratively recalculated while progressively reducing the price of cadonilimab to determine the threshold at which CAD-CHM becomes cost-effective.

### 2.6 Scenario analysis

Two scenario analyses were performed for the overall population. In Scenario 1, the model duration was adjusted to 2, 4, and 6 years to assess its influence on model outcomes. In Scenario 2, it was assumed that only 30% or 50% of patients received the best supportive care after disease progression, simulating real-world scenarios where some patients discontinue treatment for various reasons.

## 3 Results

### 3.1 Results of the base case analysis

In the overall population, CAD-CHM incurred a total cost of $36,207.12 and yielded 1.25 QALYs, while PLA-CHM had a total cost of $10,248.88 and provided 0.86 QALYs, resulting in an ICER of $67,378.09 per QALY. In the PD-L1 CPS ≥ 5 subgroup, CAD-CHM cost $39,098.19 and achieved 1.46 QALYs, whereas PLA-CHM cost $10,301.32 and yielded 0.87 QALYs, with an ICER of $48,433.34 per QALY. In the PD-L1 CPS < 5 subgroup, CAD-CHM incurred a total cost of $33,824.19 and provided 1.17 QALYs, while PLA-CHM cost $10,334.52 and generated 0.87 QALYs, resulting in an ICER of $78,463.86 per QALY (**Table 3**). All ICERs exceeded the WTP threshold of $41,511 per QALY, indicating that CAD-CHM is not cost-effective compared with chemotherapy alone for HER2-negative advanced G/GEJ adenocarcinoma from the perspective of the Chinese healthcare system.

**Table 3:** The cost and outcome results of the base-case analysis.

Parameters	Overall population		PD-L1 CPS ≥ 5 subgroup		PD-L1 CPS < 5 subgroup	
	CAD-CHM group	PLA-CHM group	CAD-CHM group	PLA-CHM group	CAD-CHM group	PLA-CHM group
Total cost ($)	36,207.12	10,248.88	39,098.19	10,301.32	33,824.19	10,334.52
Incremental costs ($)	25,958.24	–	28,793.87	–	23,489.67	–
Total effectiveness (QALYs)	1.25	0.86	1.46	0.87	1.17	0.87
Incremental effectiveness (QALYs)	0.39	–	0.59	–	0.30	–
ICER ($/QALY)	67,378.09	–	48,433.34	–	78,463.86	–

CAD-CHM, cadonilimab plus chemotherapy; CPS, combined positive score; ICER, incremental cost-effectiveness ratio; OS, overall survival; PD, progressive disease; PD-L1, programmed death ligand 1; PFS, progression-free survival; PLA-CHM, placebo plus chemotherapy; QALY, quality-adjusted life year.

### 3.2 Sensitivity analysis

**Figure 2:**
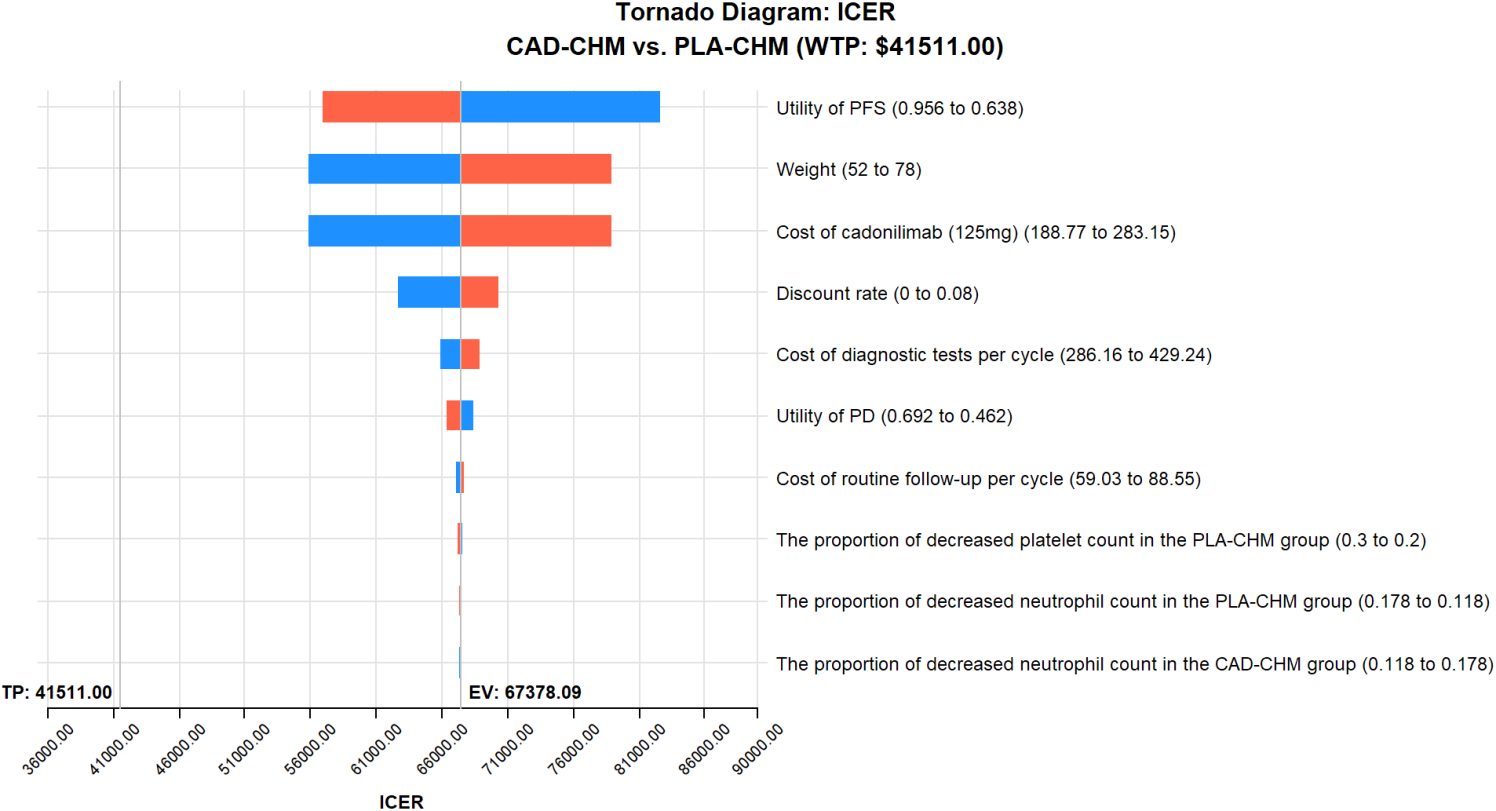
One-way sensitivity analyses in the overall population. CAD-CHM, cadonilimab plus chemotherapy; ICER, incremental cost-effectiveness ratio; PD, progressive disease; PFS, progression-free survival; PLA-CHM, placebo plus chemotherapy.

**Figure 3:**
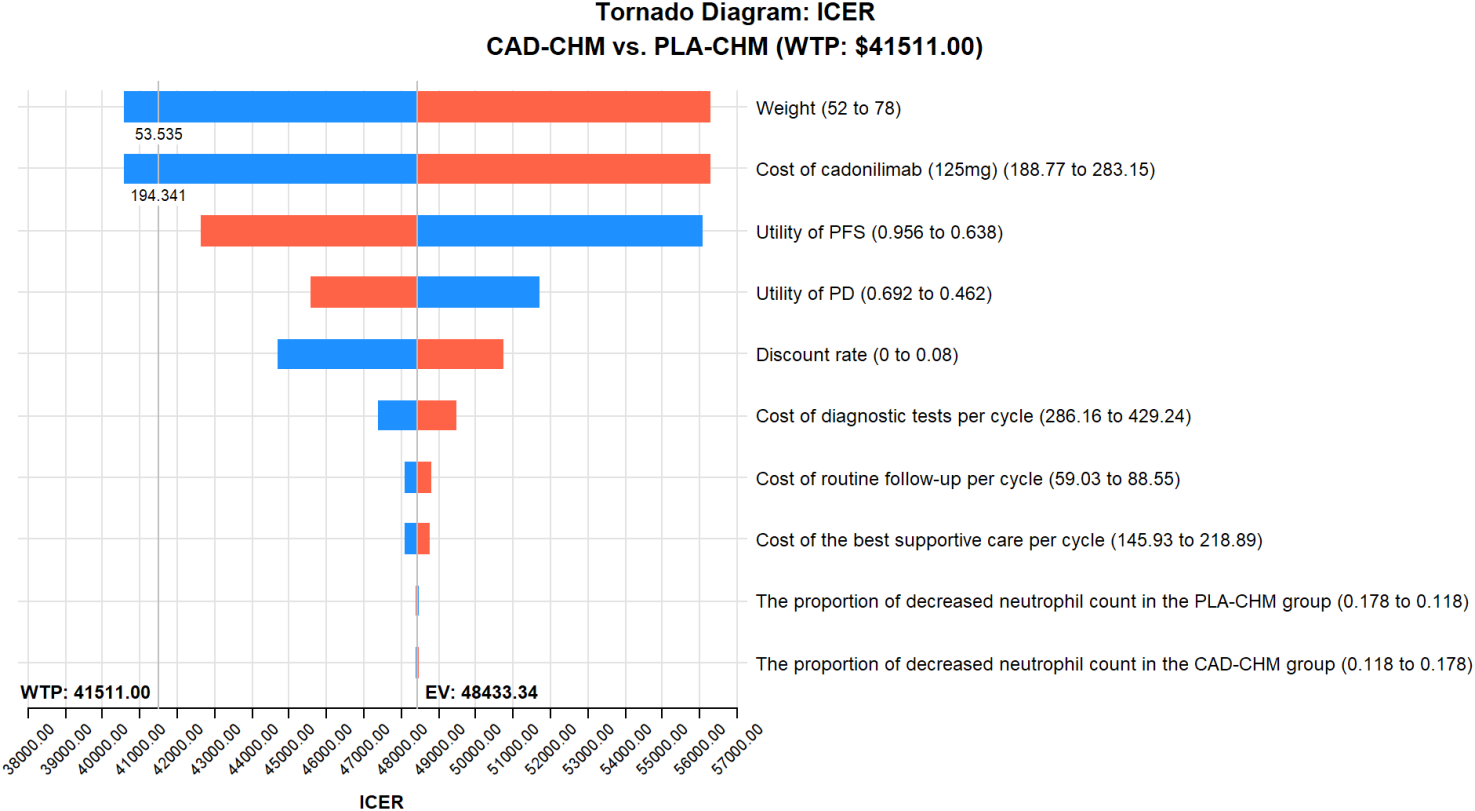
One-way sensitivity analyses in PD-L1 CPS ≥ 5 subgroup. CAD-CHM, cadonilimab plus chemotherapy; ICER, incremental cost-effectiveness ratio; PD, progressive disease; PFS, progression-free survival; PLA-CHM, placebo plus chemotherapy.

**Figure 4:**
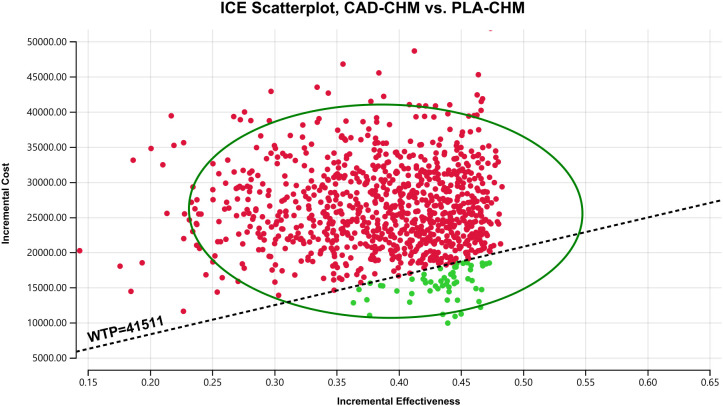
One-way sensitivity analyses in the PD-L1 CPS < 5 subgroup. CAD-CHM, cadonilimab plus chemotherapy; ICER, incremental cost-effectiveness ratio; PD, progressive disease; PFS, progression-free survival; PLA-CHM, placebo plus chemotherapy.

**Figure 5:**
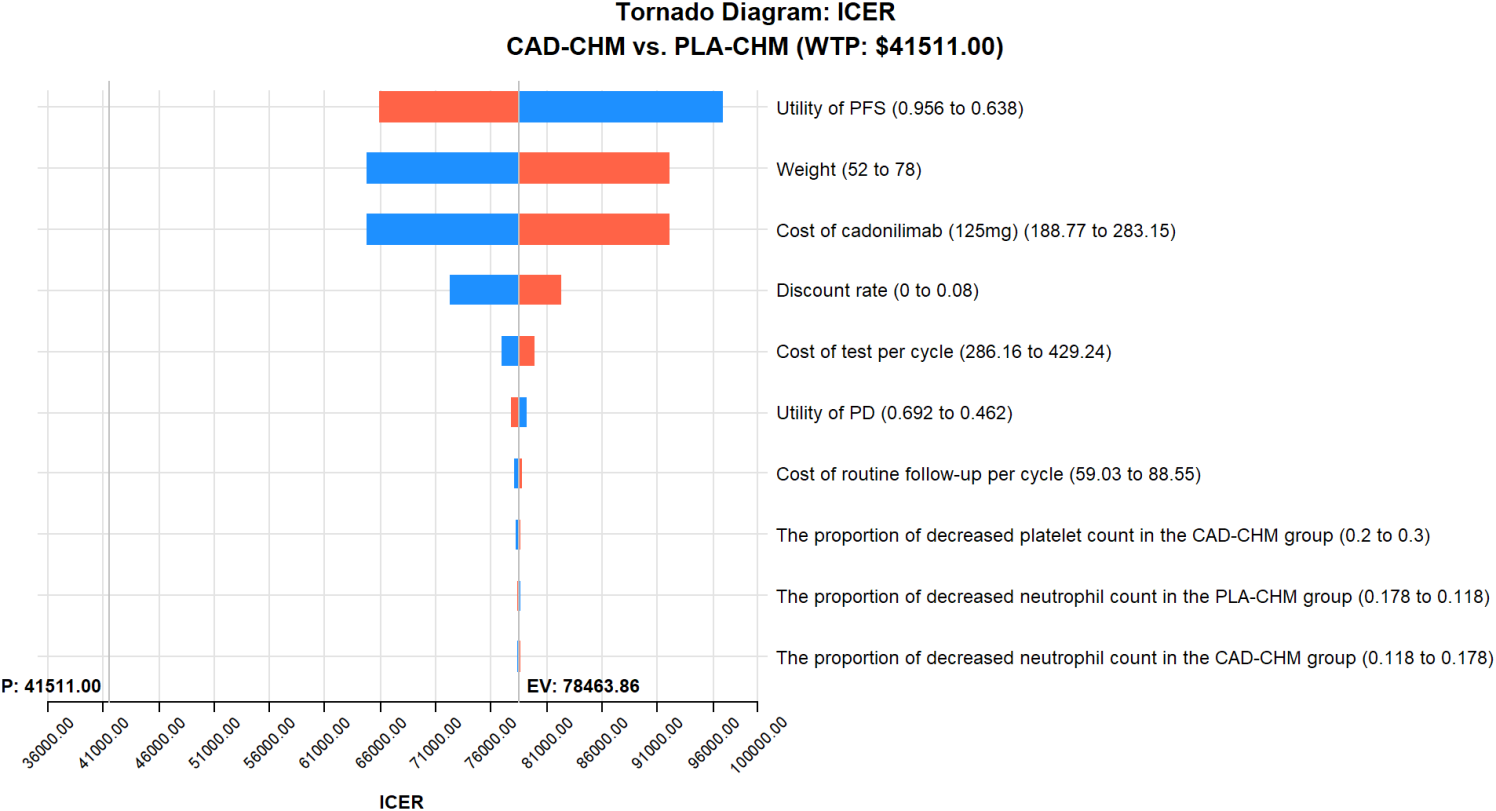
Probabilistic sensitivity analysis in the overall population. CAD-CHM, cadonilimab plus chemotherapy; ICE, incremental cost-effectiveness; PLA-CHM, placebo plus chemotherapy; WTP, willingness-to-pay.

**Figure 6:**
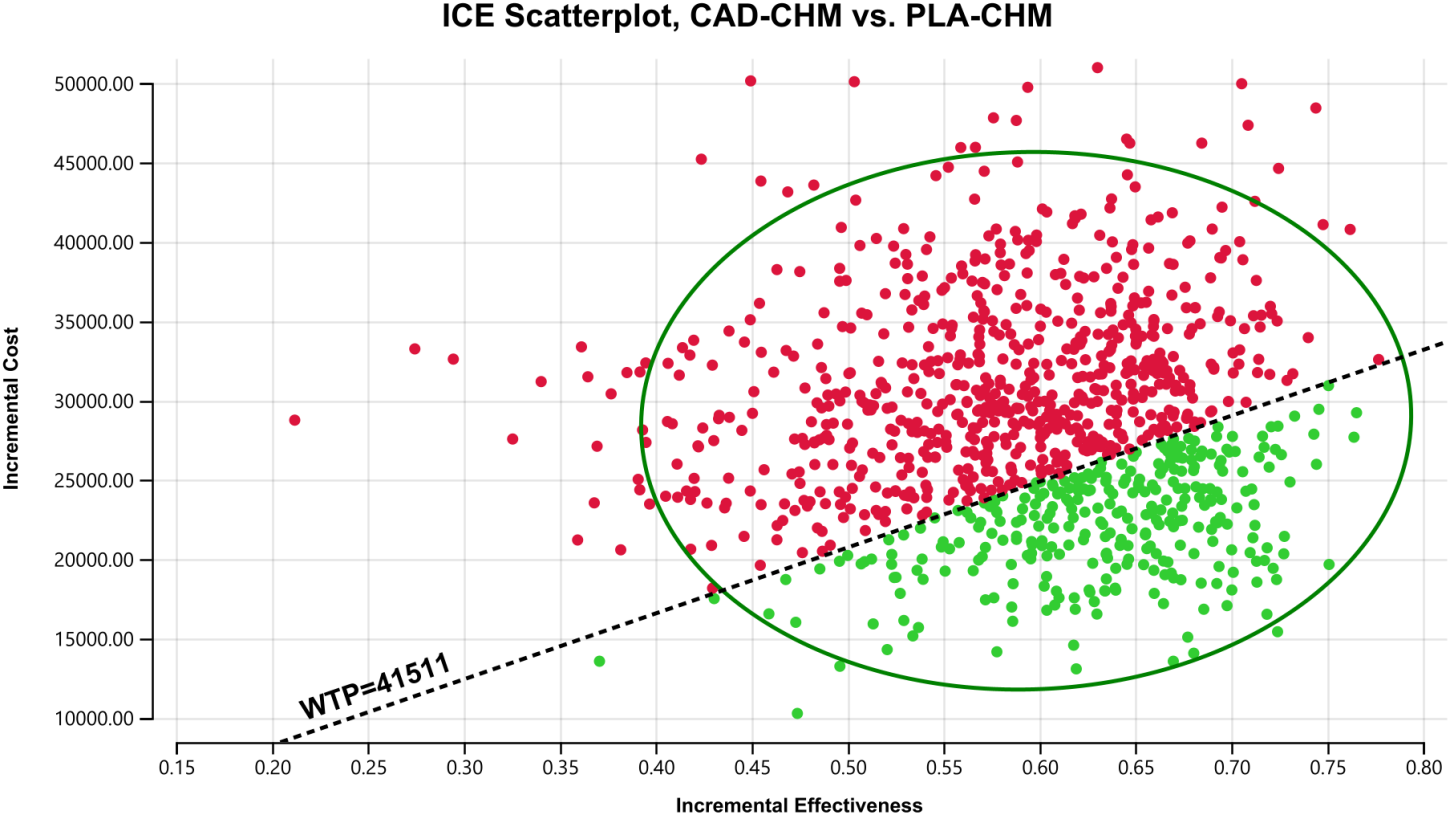
Probabilistic sensitivity analysis in PD-L1 CPS ≥ 5 subgroup. CAD-CHM, cadonilimab plus chemotherapy; ICE, incremental cost-effectiveness; PLA-CHM, placebo plus chemotherapy; WTP, willingness-to-pay.

**Figure 7:**
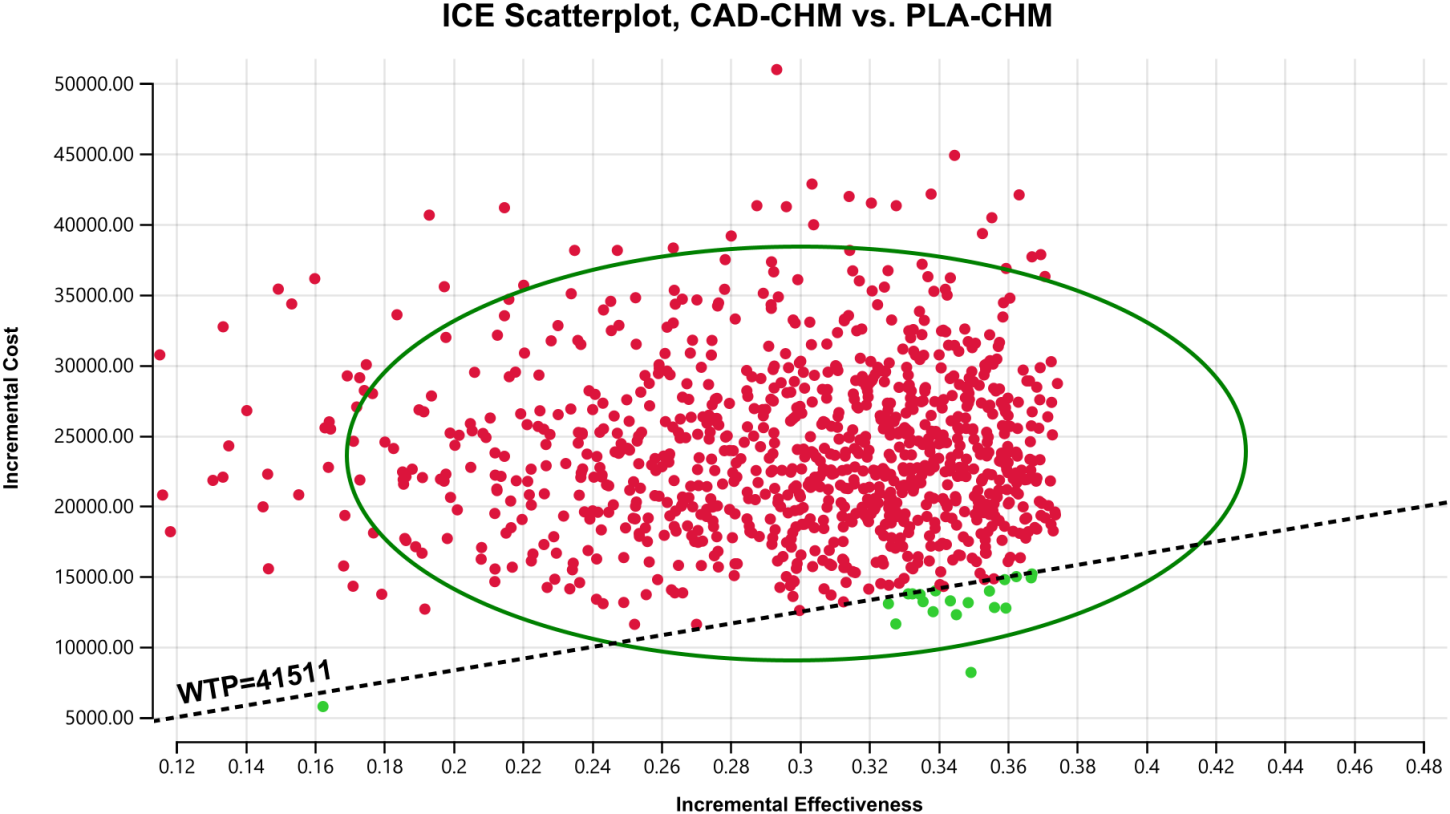
Probabilistic sensitivity analysis in PD-L1 CPS < 5 subgroup. CAD-CHM, cadonilimab plus chemotherapy; ICE, incremental cost-effectiveness; PLA-CHM, placebo plus chemotherapy; WTP, willingness-to-pay.

One-way sensitivity analysis results, visualized in a tornado diagram (**Figures 2**–**4**), indicated that patient weight, cadonilimab cost, and PFS utility value were the most influential parameters in the overall population and the PD-L1 CPS < 5 subgroup. However, even under parameter variations, the ICER remained above the WTP threshold, suggesting minimal impact on model conclusions. In contrast, in the PD-L1 CPS ≥ 5 subgroup, CAD-CHM became cost-effective when patient weight and cadonilimab cost approached their lower limits. Probabilistic sensitivity analysis results, illustrated in a scatter plot (**Figures 5**–**7**), showed that the probability of CAD-CHM being cost-effective at a WTP threshold of $41,511 per QALY was 6.4% in the overall population, 31.0% in the PD-L1 CPS ≥ 5 subgroup, and 2.4% in the PD-L1 CPS < 5 subgroup. Additionally, cost-reduction analysis revealed that CAD-CHM would only become a cost-effective first-line option for HER2-negative advanced G/GEJ adenocarcinoma in the overall population if the cost of cadonilimab (150 mg) dropped below $129.5.

### 3.3 Scenario analysis

Scenario analysis results are presented in **Table 4**. In Scenario 1, when the model duration was adjusted to 2, 4, and 6 years, the ICERs of CAD-CHM compared with PLA-CHM were $115,126.94/QALY, $81,383.15/QALY, and $72,632.12/QALY, respectively. This indicates a progressive decline in ICER with increasing model duration. In Scenario 2, when the proportion of patients receiving the best supportive care was set at 30% and 50%, the ICERs of CAD-CHM compared with PLA-CHM were $66,971/QALY and $67,087.94/QALY, respectively. This indicates that the model results are relatively insensitive to assumptions regarding changes in the proportion of patients receiving the best supportive care.

**Table 4:** Scenario analyses in the overall population.

Scenarios	Cost ($)		QALY		ICER ($/QALY)
	CAD-CHM group	PLA-CHM group	CAD-CHM group	PLA-CHM group	
Scenario 1					
Model runtime (year) =2	28,851.56	9,018.77	0.88	0.71	115,126.94
Model runtime (year) =4	33,334.83	9,824.75	1.10	0.81	81,383.15
Model runtime (year) =6	35,029.39	10,085.97	1.18	0.84	72,632.12
Scenario 2					
30% of patients receive BSC	33,935.08	8,133.34	1.25	0.86	66,971.88
50% of patients receive BSC	34,584.24	8,737.78	1.25	0.86	67,087.94

BSC, best supportive care; CAD-CHM, cadonilimab plus chemotherapy; CPS, combined positive score; ICER, incremental cost-effectiveness ratio; PLA-CHM, placebo plus chemotherapy; QALY, quality-adjusted life year.

## 4 Discussion

Co-inhibition of PD-1 and CTLA-4 induces a synergistic anti-tumor response by reshaping the tumor immune microenvironment into a more immunogenic phenotype (42). The complementary effects of CTLA-4 and PD-1 inhibitors have been well established (43). Cadonilimab, the first bispecific immune checkpoint inhibitor approved globally, simultaneously targets PD-1 and CTLA-4, enhancing anti-tumor efficacy through dual-pathway blockade (18). The COMPASSION-15 trial (21) demonstrated that CAD-CHM significantly improves survival in patients with HER2-negative advanced G/GEJ adenocarcinoma, underscoring its clinical potential. However, the escalating costs of novel cancer therapies pose a substantial challenge to healthcare system sustainability. As previously reported (23, 44), comprehensive cost-effectiveness evaluations are crucial for guiding policy decisions and optimizing healthcare resource allocation. Given the high cost of CAD-CHM, a thorough assessment of its cost-effectiveness as a first-line treatment for HER2-negative advanced G/GEJ adenocarcinoma is imperative to ensure both economic feasibility and equitable access within resource-constrained healthcare systems.

This study represents the first cost-effectiveness analysis of CAD-CHM as a first-line treatment for HER2-negative advanced G/GEJ adenocarcinoma within the Chinese healthcare system. Beyond its domestic significance, the findings offer valuable insights for the global medical community, marking a core innovation of this research. Compared with PLA-CHM, the incremental cost per additional QALY gained with CAD-CHM amounts to $67,378.09 in the overall population, $48,433.34 in the PD-L1 CPS ≥ 5 subgroup, and $78,463.86 in the PD-L1 CPS < 5 subgroup—substantially exceeding the predefined WTP threshold of $41,511 per QALY. Consequently, CAD-CHM does not demonstrate cost-effectiveness as a first-line therapy for HER2-negative advanced G/GEJ adenocarcinoma, irrespective of PD-L1 CPS status, from the perspective of the Chinese healthcare system. This outcome likely stems from the prolonged maintenance therapy required for cadonilimab and its substantially higher cost relative to oxaliplatin and capecitabine, leading to significantly elevated drug expenditures without providing sufficient incremental survival benefits. One-way sensitivity analysis identified patient weight (which determines cadonilimab dosing) and drug cost as the most influential factors in the cost-effectiveness of CAD-CHM, further supporting this conclusion. These findings highlight an urgent need to reduce the cost of cadonilimab to improve the affordability of the CAD-CHM regimen. Policy interventions should be implemented to enhance the cost-effectiveness of these promising treatments, ensuring broader patient access. Pharmaceutical companies can mitigate costs by optimizing manufacturing processes, improving supply chain efficiency, and refining pricing strategies, thereby enhancing the economic viability of this regimen and facilitating the widespread adoption of innovative therapies. Additionally, the analysis suggests that the cost of cadonilimab (125 mg) must be reduced to below 54.89% of its current price—specifically, under $129.50—for CAD-CHM to become a cost-effective first-line option for HER2-negative advanced G/GEJ adenocarcinoma in the overall population. This threshold provides a critical pricing benchmark for both healthcare policymakers and pharmaceutical manufacturers.

Subgroup analysis revealed that the ICER in the PD-L1 CPS ≥ 5 subgroup was substantially lower than that in the overall population, whereas the ICER in the PD-L1 CPS < 5 subgroup exceeded that of the overall population. Although neither subgroup achieved cost-effectiveness, the CAD-CHM regimen demonstrated greater economic viability in the PD-L1 CPS ≥ 5 subgroup. This underscores the critical role of PD-L1 expression level detection, which may serve as a strategy to enhance the cost-effectiveness of CAD-CHM in treating HER2-negative advanced G/GEJ adenocarcinoma. These findings provide valuable guidance for Chinese medical insurance policymakers in defining appropriate reimbursement criteria for cadonilimab.

Scenario analysis has proven instrumental in evaluating drug cost-effectiveness by accounting for varying assumptions and uncertainties, thereby better approximating real-world complexities. Accordingly, two scenario analyses were conducted. Scenario 1 demonstrated that prolonged treatment duration improves the cost-effectiveness of CAD-CHM. Scenario 2 indicated that following disease progression, an increased proportion of patients receiving the best supportive care does not substantially impact the ICER of CAD-CHM, suggesting that continued supportive care does not significantly diminish the cost-effectiveness of CAD-CHM. These analyses suggest that extended treatment adherence may optimize therapeutic value, aligning with the interests of clinicians, patients, and their families, as well as broader ethical and societal considerations.

To date, only two cost-effectiveness studies have assessed the use of immune checkpoint inhibitors as first-line treatments for HER2-negative advanced G/GEJ adenocarcinoma within the Chinese healthcare system. Lang et al. (45) and Zhang et al. (46) concluded that pembrolizumab plus chemotherapy is not cost-effective as a first-line option for treating HER2-negative advanced G/GEJ adenocarcinoma. These findings are consistent with the present study, which similarly identified no economic advantage of CAD-CHM over chemotherapy alone.

This study possesses several notable strengths. First, it leverages the most recent data from the COMPASSION-15 trial, incorporating nearly two years of survival analysis to compare the efficacy of cadonilimab plus chemotherapy with chemotherapy alone, thereby providing the latest clinical evidence. Second, as all participants in the COMPASSION-15 trial were Chinese patients, the findings exhibit strong population-specific applicability, offering a more accurate reflection of treatment outcomes and economic benefits within the Chinese healthcare system. Lastly, through comprehensive subgroup and scenario analyses, the study evaluates economic impacts across diverse patient cohorts and treatment conditions, furnishing critical insights for clinicians, patients, and policymakers in making personalized treatment decisions.

However, certain limitations should be acknowledged. First, given that the COMPASSION-15 trial remains ongoing, long-term survival data are currently unavailable. This study extrapolated survival beyond the follow-up period using survival models, which may introduce some deviation from actual outcomes. For instance, the tail of the survival curve for patients receiving immunotherapy may exhibit a plateau (47). Our model does not account for the possibility of long-term survival and may therefore underestimate the efficacy of immunotherapy. Future studies should validate these findings using real-world data for cost-effectiveness analysis. Second, post-progression treatment details were not reported in the COMPASSION-15 trial, necessitating the assumption that all patients received the best supportive care after first-line treatment failure, which may not fully align with real-world clinical practice. In reality, the selection of subsequent treatment regimens is individualized based on each patient’s specific circumstances. Fortunately, the results of the one-way sensitivity analysis provided reassurance, as they consistently indicated that altering the estimated range of subsequent treatments would not change the model’s outcomes. Third, due to the absence of quality-of-life data in the trial, health utility values were sourced from Chinese literature, potentially introducing bias into the model; however, sensitivity analysis confirmed that this does not alter the study’s overall conclusions. However, it must be acknowledged that obtaining more accurate health utility values is crucial for enhancing the accuracy of our model outcomes. Should future clinical studies report health-related quality-of-life outcomes specific to the Chinese population, incorporating these reliable data would optimize our model results. Lastly, this analysis focused solely on the cost-effectiveness of CAD-CHM relative to chemotherapy alone, without considering alternative treatment regimens such as pembrolizumab plus chemotherapy, owing to the lack of direct comparative data. However, the studies by Lang et al. (45) and Zhang et al. (46) suggest that pembrolizumab combined with chemotherapy is not cost-effective compared to chemotherapy alone. Therefore, we believe that selecting chemotherapy as the comparator in the cost-effectiveness analysis of CAD-CHM is reasonable. Despite these limitations, the findings remain highly informative for healthcare policymakers, clinicians, and patients.

## 5 Conclusion

The study results indicate that, from the perspective of the Chinese healthcare system, CAD-CHM as a first-line treatment for HER2-negative advanced G/GEJ adenocarcinoma lacks cost-effectiveness compared with chemotherapy alone, irrespective of PD-L1 CPS subgroup stratification.

## Data Availability

The original contributions presented in the study are included in the article/**Supplementary Material**. Further inquiries can be directed to the corresponding author.
