# Supplementary material for: Cost-effectiveness analysis of first-line cadonilimab plus chemotherapy in HER2-negative advanced gastric or gastroesophageal junction adenocarcinoma
Source: Front Immunol. 2025 May 13;16:1575627. doi: 10.3389/fimmu.2025.1575627 (PMC12106304; doi:10.3389/fimmu.2025.1575627)
Supplement: Supplementary file 1 [file DataSheet1.docx]

Supplementary Material

**Cost-Effectiveness Analysis of First-line Cadonilimab Plus Chemotherapy in HER2-Negative Advanced Gastric or Gastroesophageal Junction Adenocarcinoma**

**1. Supplementary Table 1. CHEERS 2022 Checklist.**

**2. Supplementary Table 2. Comparison of survival models.**

**3. Supplementary Figure 1. Results of the survival curve fit the CAD-CHM group and PLA-CHM group.**

1. **Supplementary Table A. CHEERS 2022 Checklist.**

| **Topic** | **No.** | **Item** | **Reported** |
| --- | --- | --- | --- |
| **Title** |  |  |  |
|  | 1 | Identify the study as an economic evaluation and specify the interventions being compared. | Yes |
| **Abstract** |  |  |  |
|  | 2 | Provide a structured summary that highlights context, key methods, results, and alternative analyses. | Yes |
| **Introduction** |  |  |  |
| **Background and objectives** | 3 | Give the context for the study, the study question, and its practical relevance for decision making in policy or practice. | Yes |
| **Methods** |  |  |  |
| **Health economic analysis plan** | 4 | Indicate whether a health economic analysis plan was developed and where available. | Yes |
| **Study population** | 5 | Describe characteristics of the study population (such as age range, demographics, socioeconomic, or clinical characteristics). | Yes |
| **Setting and location** | 6 | Provide relevant contextual information that may influence findings. | Yes |
| **Comparators** | 7 | Describe the interventions or strategies being compared and why chosen. | Yes |
| **Perspective** | 8 | State the perspective(s) adopted by the study and why chosen. | Yes |
| **Time horizon** | 9 | State the time horizon for the study and why appropriate. | Yes |
| **Discount rate** | 10 | Report the discount rate(s) and reason chosen. | Yes |
| **Selection of outcomes** | 11 | Describe what outcomes were used as the measure(s) of benefit(s) and harm(s). | Yes |
| **Measurement of outcomes** | 12 | Describe how outcomes used to capture benefit(s) and harm(s) were measured. | Yes |
| **Valuation of outcomes** | 13 | Describe the population and methods used to measure and value outcomes. | Yes |
| **Measurement and valuation of resources and costs** | 14 | Describe how costs were valued. | Yes |
| **Currency, price date, and conversion** | 15 | Report the dates of the estimated resource quantities and unit costs, plus the currency and year of conversion. | Yes |
| **Rationale and description of model** | 16 | If modelling is used, describe in detail and why used. Report if the model is publicly available and where it can be accessed. | Yes |
| **Analytics and assumptions** | 17 | Describe any methods for analysing or statistically transforming data, any extrapolation methods, and approaches for validating any model used. | Yes |
| **Characterising heterogeneity** | 18 | Describe any methods used for estimating how the results of the study vary for subgroups. | Yes |
| **Characterising distributional effects** | 19 | Describe how impacts are distributed across different individuals or adjustments made to reflect priority populations. | Yes |
| **Characterising uncertainty** | 20 | Describe methods to characterise any sources of uncertainty in the analysis. | Yes |
| **Approach to engagement with patients and others affected by the study** | 21 | Describe any approaches to engage patients or service recipients, the general public, communities, or stakeholders (such as clinicians or payers) in the design of the study. | Yes |
| **Results** |  |  |  |
| **Study parameters** | 22 | Report all analytic inputs (such as values, ranges, references) including uncertainty or distributional assumptions. | Yes |
| **Summary of main results** | 23 | Report the mean values for the main categories of costs and outcomes of interest and summarise them in the most appropriate overall measure. | Yes |
| **Effect of uncertainty** | 24 | Describe how uncertainty about analytic judgments, inputs, or projections affect findings. Report the effect of choice of discount rate and time horizon, if applicable. | Yes |
| **Effect of engagement with patients and others affected by the study** | 25 | Report on any difference patient/service recipient, general public, community, or stakeholder involvement made to the approach or findings of the study | Yes |
| **Discussion** |  |  |  |
| **Study findings, limitations, generalisability, and current knowledge** | 26 | Report key findings, limitations, ethical or equity considerations not captured, and how these could affect patients, policy, or practice. | Yes |
| **Other relevant information** |  |  |  |
| **Source of funding** | 27 | Describe how the study was funded and any role of the funder in the identification, design, conduct, and reporting of the analysis | Yes |
| **Conflicts of interest** | 28 | Report authors conflicts of interest according to journal or International Committee of Medical Journal Editors requirements. | Yes |

1. **Supplementary Table 2. Comparison of survival models.**

|  | AIC | | BIC | | |
| --- | --- | --- | --- | --- | --- |
|  | CAD-CHM group | PLA-CHM group | CAD-CHM group | PLA-CHM group | |
| OS-overall population | | |  |  | |
| Exponential | 1340.992 | 1558.703 | 1344.712 | 1562.423 | |
| Gamma | 1320.466 | 1517.398 | 1327.907 | 1524.839 | |
| Gen.F | 1317.248 | 1520.859 | 1332.129 | 1535.740 | |
| Gen.Gamma | 1318.249 | 1518.986 | 1326.409 | 1530.147 | |
| Gompertz | 1336.280 | 1535.948 | 1343.721 | 1543.388 | |
| Weibull | 1324.075 | 1520.044 | 1331.516 | 1527.484 | |
| Log-logistic | 1316.341 | 1518.063 | 1323.781 | 1525.503 | |
| Log-normal | 1318.273 | 1523.897 | 1326.713 | 1531.337 | |
| PFS-overall population | |  |  |  | |
| Exponential | 700.748 | 1461.344 | 703.738 | 1465.064 | |
| Gamma | 679.188 | 1404.153 | 685.169 | 1411.594 | |
| Gen.F | 667.194 | 1395.620 | 679.155 | 1410.501 | |
| Gen.Gamma | 665.192 | 1393.598 | 674.163 | 1404.758 | |
| Gompertz | 701.029 | 1450.829 | 707.010 | 1458.270 | |
| Weibull | 686.988 | 1417.337 | 692.969 | 1424.778 | |
| Log-logistic | 664.541 | 1392.101 | 672.522 | 1401.542 | |
| Log-normal | 670.366 | 1402.857 | 675.954 | 1407.425 | |
| OS-PD-L1 CPS ≥ 5 subgroup | | |  |  | |
| Exponential | 437.971 | 695.221 | 440.724 | 698.162 | |
| Gamma | 436.245 | 679.983 | 441.752 | 685.866 | |
| Gen.F | 436.710 | 683.326 | 447.724 | 695.092 | |
| Gen.Gamma | 434.710 | 681.981 | 442.970 | 690.806 | |
| Gompertz | 439.588 | 686.575 | 445.095 | 692.458 | |
| Weibull | 437.056 | 680.571 | 442.563 | 686.454 | |
| Log-logistic | 430.663 | 680.713 | 440.171 | 686.596 | |
| Log-normal | 433.024 | 684.815 | 438.532 | 690.698 | |
| PFS- PD-L1 CPS ≥ 5 subgroup | | |  |  | |
| Exponential | 1461.344 | 678.789 | 1465.064 | 681.731 | |
| Gamma | 1404.153 | 644.154 | 1411.594 | 650.037 | |
| Gen.F | 1395.620 | 646.208 | 1410.501 | 657.975 | |
| Gen.Gamma | 1393.598 | 645.530 | 1404.758 | 654.355 | |
| Gompertz | 1450.829 | 664.320 | 1458.270 | 670.204 | |
| Weibull | 1417.337 | 647.856 | 1424.778 | 653.740 | |
| Log-logistic | 1390.101 | 643.210 | 1398.542 | 651.093 | |
| Log-normal | 1391.775 | 646.916 | 1399.216 | 652.799 | |
| OS-PD-L1 CPS < 5 subgroup | | |  |  |  |
| Exponential | 729.746 | 749.853 | 732.802 | 752.844 | |
| Gamma | 717.459 | 724.704 | 723.572 | 730.685 | |
| Gen.F | 717.904 | 727.407 | 730.129 | 739.369 | |
| Gen.Gamma | 715.903 | 725.404 | 725.071 | 734.375 | |
| Gompertz | 726.574 | 737.071 | 732.687 | 743.052 | |
| Weibull | 719.552 | 727.089 | 725.665 | 733.070 | |
| Log-logistic | 712.602 | 722.387 | 719.715 | 727.368 | |
| Log-normal | 713.903 | 723.951 | 720.015 | 729.932 | |
| PFS-PD-L1 CPS < 5 subgroup | | |  | |  |
| Exponential | 688.319 | 700.748 | 691.375 | 703.738 | |
| Gamma | 674.478 | 679.188 | 680.591 | 685.169 | |
| Gen.F | 671.104 | 667.194 | 683.329 | 679.155 | |
| Gen.Gamma | 669.100 | 665.192 | 678.269 | 674.163 | |
| Gompertz | 686.882 | 701.029 | 692.994 | 707.010 | |
| Weibull | 678.095 | 686.988 | 684.208 | 692.969 | |
| Log-logistic | 666.879 | 664.514 | 675.991 | 672.522 | |
| Log-normal | 667.295 | 664.918 | 676.408 | 673.899 | |

AIC, Akaike information criterion; BIC, Bayesian Information Criterion; CAD-CHM, cadonilimab plus chemotherapy; CPS, combined positive score; OS, overall survival; OS, overall survival; PD-L1, programmed death ligand 1; PFS, progression-free survival; PLA-CHM, placebo plus chemotherapy.

**3.** **Supplementary Figure 1. Results of the survival curve fit the CAD-CHM group and PLA-CHM group.**

**
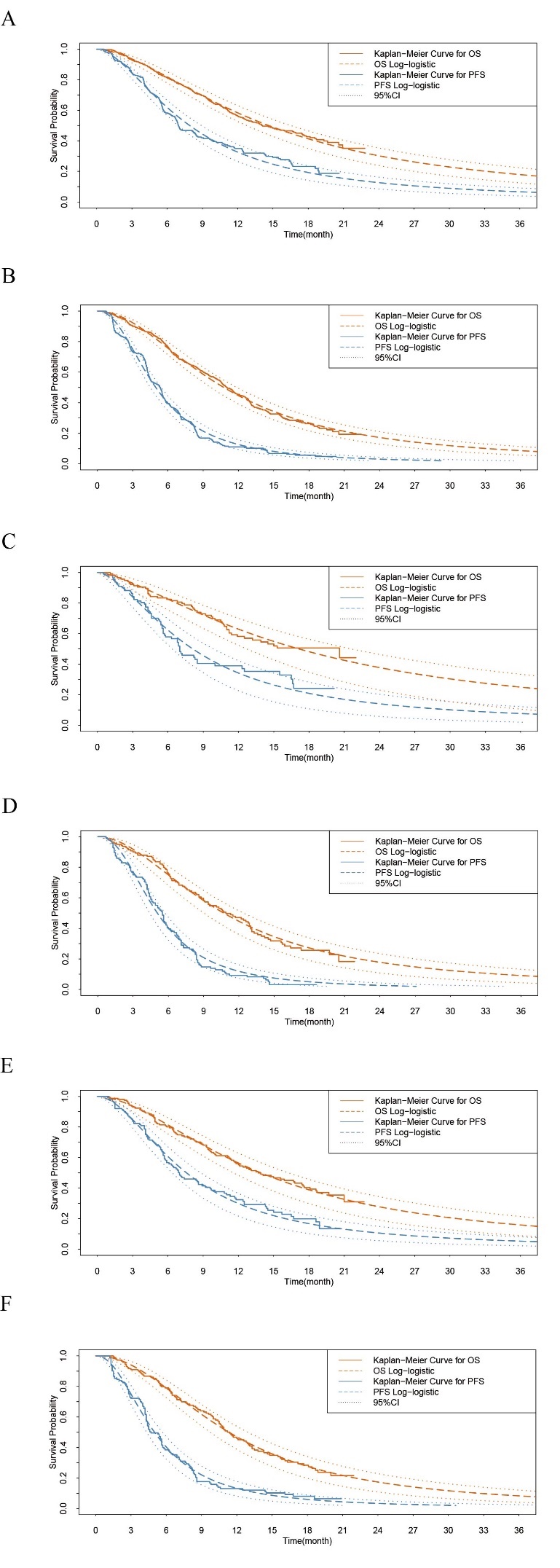
**

A, C, and E were survival curve fitting results in the CAD-CHM group for patients with the overall population, PD-L1 CPS ≥ 5 subgroup, and PD-L1 CPS < 5 subgroup, respectively. B, D, and F were survival curve fitting results in the PLA-CHM group for patients with the overall population, PD-L1 CPS ≥ 5 subgroup, and PD-L1 CPS < 5 subgroup, respectively. PFS, progression-free survival; OS, overall survival.
